# Localized gastric amyloidosis differentiated histologically from scirrhous gastric cancer using endoscopic mucosal resection: a case report

**DOI:** 10.1186/1752-1947-6-231

**Published:** 2012-08-03

**Authors:** Tsugumasa Kamata, Haruhisa Suzuki, Shigetaka Yoshinaga, Satoru Nonaka, Takeo Fukagawa, Hitoshi Katai, Hirokazu Taniguchi, Ryoji Kushima, Ichiro Oda

**Affiliations:** 1 Endoscopy Division, National Cancer Center Hospital, 5-1-1 Tsukiji, Chuo-ku, Tokyo, 104-0045, Japan; 2 Gastric Surgery Division, National Cancer Center Hospital, 5-1-1 Tsukiji, Chuo-ku, Tokyo, 104-0045, Japan; 3 Pathology Division, National Cancer Center Hospital, 5-1-1 Tsukiji, Chuo-ku, Tokyo, 104-0045, Japan

**Keywords:** Endoscopic mucosal resection (EMR), Localized gastric amyloidosis, Scirrhous gastric cancer

## Abstract

**Introduction:**

Amyloidosis most often manifests as a systemic involvement of multiple tissues and organs, and an amyloidal deposit confined to the stomach is extremely rare. It is sometimes difficult to provide a definitive diagnosis of localized gastric amyloidosis by biopsy specimen and diagnosis of amyloidosis in some cases has been finalized only after surgical resection of the stomach.

**Case presentation:**

A 76-year-old Japanese woman with epigastric discomfort underwent an esophagogastroduodenoscopy procedure. The esophagogastroduodenoscopy revealed gastric wall thickening, suggesting scirrhous gastric carcinoma, at the greater curvature from the upper to the lower part of the gastric corpus. A biopsy specimen revealed amyloid deposits in the submucosal layer with no malignant findings. We resected a representative portion of the lesion by endoscopic mucosal resection using the strip biopsy method to obtain sufficient tissue specimens, and then conducted a detailed histological evaluation of the samples. The resected specimens revealed deposition of amyloidal materials in the gastric mucosa and submucosa without any malignant findings. Congo red staining results were positive for amyloidal protein and exhibited green birefringence under polarized light. Congo red staining with prior potassium permanganate incubation confirmed the light chain (AL) amyloid protein type. Based on these results, gastric malignancy, systemic amyloidosis and amyloid deposits induced by inflammatory disease were excluded and this lesion was consequently diagnosed as localized gastric amyloidosis. Our patient was an older woman and there were no findings relative to an increase in gastrointestinal symptoms or anemia, so no further treatment was performed. She continued to be in good condition without any finding of disease progression six years after verification of our diagnosis.

**Conclusions:**

We report an unusual case of primary amyloidosis of the stomach resembling scirrhous gastric carcinoma. This case of localized gastric amyloidosis was differentiated from scirrhous gastric cancer after performing endoscopic mucosal resection without an invasive surgical resection, as endoscopic mucosal resection provided sufficient tissue specimens from the lesion to make an accurate histological evaluation.

## Introduction

The term amyloidosis refers to a group of disorders characterized by extracellular accumulation of insoluble, fibrillar proteins in various organs and tissues. Amyloidosis most often manifests as systemic involvement of multiple tissues and organs, and an amyloidal deposit confined to the stomach has been extremely rare in previously published reports. It is sometimes difficult to provide a definitive diagnosis of localized gastric amyloidosis by biopsy specimen and diagnosis of amyloidosis in some cases was definitively made only after surgical resection of the stomach.

## Case presentation

A 76-year-old Japanese woman presented to our facility with epigastric discomfort; she had previously undergone an esophagogastroduodenoscopy (EGD) procedure at another hospital. The EGD had revealed gastric wall thickening, suggesting scirrhous gastric carcinoma, so our patient was referred to our hospital for further examination and treatment.

**Figure 1:**
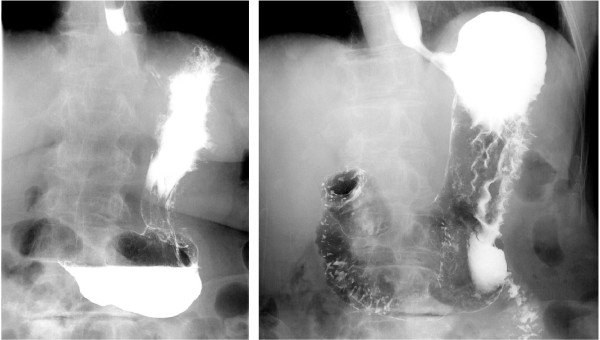
**Barium upper gastrointestinal X-ray series findings.** Barium upper gastrointestinal X-ray series indicating rigidity and poor extensibility of the gastric wall from the upper to lower gastric body.

**Figure 2:**
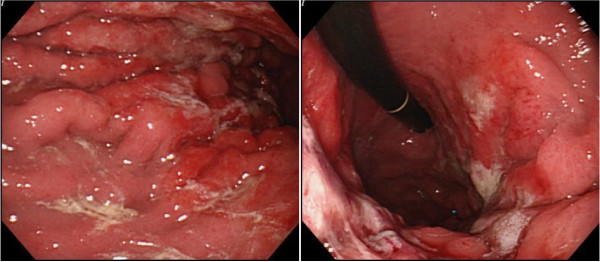
**Esophagogastroduodenoscopy findings.** Esophagogastroduodenoscopy revealing reddish and markedly swollen folds with erosions at the greater curvature from the upper to lower part of the gastric corpus.

**Figure 3:**
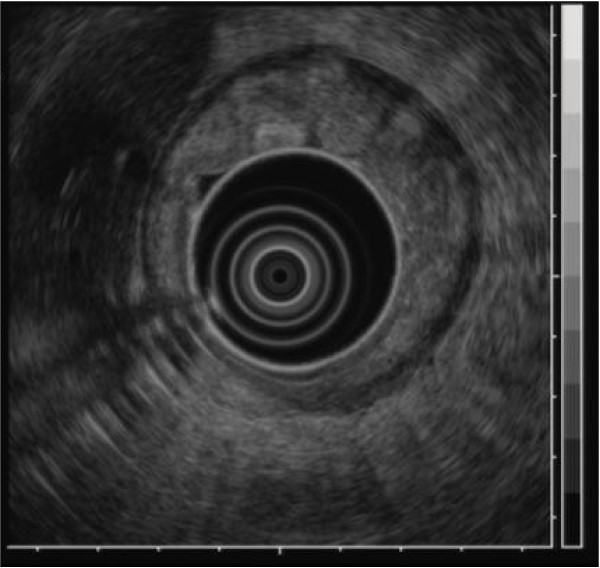
**Endoscopic ultrasound findings.** Endoscopic ultrasound showing obvious thickening of the submucosal layer without disruption of the gastric wall structure.

A barium upper gastrointestinal X-ray series indicated rigidity and poor extensibility of the gastric wall from the fornix to the lower gastric body, and irregular, enlarged folds were also noticeable (Figure 1). EGD revealed reddish and markedly swollen folds at the greater curvature from the upper to the lower part of the gastric corpus (Figure 2). The mucosa surface was hemorrhagic and erosive. A biopsy specimen revealed amyloid deposits in the submucosal layer with no malignant findings. Endoscopic ultrasound showed obvious thickening of the third layer corresponding to the submucosal layer with no disruption of the gastric wall structure (Figure 3). Computed tomography (CT) revealed thickening of the gastric wall and enlarged circumambient lymph nodes. Biopsy specimens from the rectum and ileum indicated no amyloid deposition. Laboratory test results were negative for Bence-Jones protein in the urine, and serum immunoglobulin levels were normal. No abnormal signs were detected on echocardiography.

**Figure 4:**
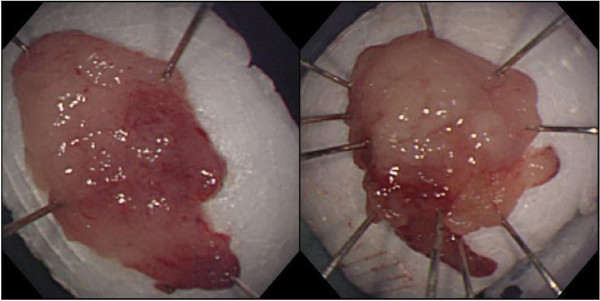
**Macroscopic findings from endoscopic mucosal resection (EMR) specimens.** Resection of representative portions of lesion by endoscopic mucosal resection was performed using the strip biopsy method.

**Figure 5:**
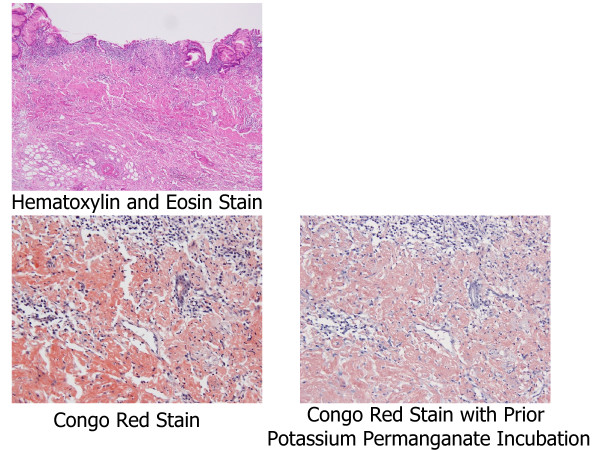
**Pathology results.** The resected specimen revealed positive deposition of light chain type amyloidal proteins from both Congo red staining and Congo red staining with prior potassium permanganate incubation.

**Figure 6:**
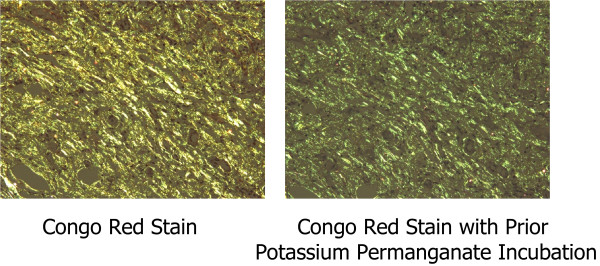
**Polarized microscopy.** Polarized microscopy showing green birefringence of deposits.

**Figure 7:**
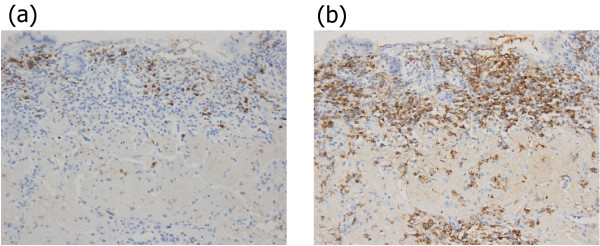
**Immunohistochemical investigation.** (**a**) κ-Chain-positive plasma cells present only in inflammatory infiltrate in the superficial lamina propria. (**b**) λ-Chain-producing plasma cells detected both in the mucosa and underlying lymphomatous proliferation.

We resected a representative portion of the lesion by endoscopic mucosal resection (EMR) using the strip biopsy method to obtain sufficient tissue specimens, and we then conducted a detailed histological evaluation (Figure 4). There were no complications associated with the EMR. The resected specimens revealed deposition of amyloidal materials in the gastric mucosa and submucosa with no malignant findings. Congo red staining results were positive for amyloidal protein and exhibited green birefringence under polarized light microscopy. Congo red staining with prior potassium permanganate incubation confirmed the light chain (AL) amyloid protein type. An immunohistochemical examination revealed that κ-chain-positive plasma cells were present only in the inflammatory infiltrate in the superficial lamina propria, while λ-chain-producing plasma cells were detected both in the mucosa and in the underlying lymphomatous proliferation (Figures 5, 6, 7). Based on these results, gastric malignancy, systemic amyloidosis and amyloid deposits induced by inflammatory disease were excluded and the lesion was consequently diagnosed as localized gastric amyloidosis. Our patient was an older woman and there were no findings relative to an increase in gastrointestinal symptoms or anemia, so no further treatment was performed and she continued to be in good condition with no findings of disease progression six years after verification of our diagnosis.

## Discussion

The term amyloidosis refers to a group of disorders characterized by extracellular accumulation of insoluble, fibrillar proteins in various organs and tissues [1]. It can result from a heterogeneous group of disorders and cause impairment or even dysfunction of involved organs [2]. Various subtypes exist, including primary systemic amyloidosis, reactive systemic amyloidosis associated with a chronic inflammatory condition and localized forms of aberrant amyloid deposition [3]. Amyloidosis most often manifests as systemic involvement of multiple tissues and organs including the heart, liver, spleen, kidneys, lymph nodes, adrenals and thyroid in addition to many others. The clinical implication of a single organ or tissue is relatively unusual and reports of an amyloidal deposit confined to the stomach are extremely rare in the previously published literature [2,3].

Clinical symptoms of localized gastric amyloidosis are often uncharacteristic and varied, including epigastric discomfort, poor appetite, hematemesis, hematochezia and gastric perforation [2,3]. In addition, the gross appearance of localized gastric amyloidosis also takes various forms such as redness, erosion, ulcer, polypoid mass, thickened folds and scirrhous-like morphology [4]. Its polypoid-mass or scirrhous-like appearance usually tends to be misdiagnosed as a gastric tumor such as gastric cancer or malignant lymphoma due to similarities in gastrointestinal symptoms and gross appearance as revealed by an endoscopic examination and other diagnostic modalities, such as a CT scan. In this respect, it has been suggested that biopsy is the only reliable means to confirm diagnosis [1,5] although it is not always possible to provide a definitive diagnosis from a biopsy specimen, and the diagnosis of amyloidosis has been finalized in some cases only after surgical resection of the stomach [6]. In particular, there have been no published case reports describing scirrhous-like localized gastric amyloidosis diagnosed by biopsy specimen that were subsequently only followed up with no surgical intervention. Therefore, the present case is the first report of scirrhous-like localized gastric amyloidosis diagnosed by a different approach than surgery. The literature review of cases of localized gastric amyloidosis is shown in Table 1.

**Table 1:** Review of the literature on cases of localized gastric amyloidosis

**Age/sex**	**Chief issue**	**Localization**	**Form**	**Amyloid protein**	**Operation**	**Prognosis**	**Author, year and reference**
55/M	Epigastric pain	Lower body	Mucosal redness	Unknown	−	Survival	Rotondano *et al*., 2007 [7]
69/F	Left flank pain	Antrum	Mucosal redness	AL	−	Died (Other disorder)	Yoshida *et al*., 1998 [8]
58/M	Appetite loss	Lower body	Depression	AA	−	Survival	Ishii *et al*., 1993 [9]
50/F	Epigastric discomfort	Lower body	Ulcer	AA	+	Survival	Wu *et al*., 2003 [2]
63/M	None	Antrum	Ulcer, tumor	AL	+	Survival (18 months)	Nishida *et al*., 1990 [10]
67/M	Appetite loss	Cardia	Tumor	AL	−	Survival	Deniz *et al*., 2006 [3]
51/F	None	Middle body to antrum	Scirrhous type	AL	+	Survival (two months)	Kato *et al*., 1988 [11]
68/F	Pain, nausea	Antrum	Scirrhous type	Unknown	+	Died (10 months)	Ikeda *et al*., 1988 [12]
56/M	Obstruction	Unknown	Scirrhous type	Unknown	+	Survival	Nfoussi *et al*., 2010 [4]
76/F	Epigastric discomfort	Upper to lower body	Scirrhous type	AL	−	Survival (six years)	Present case, 2012

With regard to diagnosis of scirrhous type gastric cancer, histological confirmation using biopsy specimens is also sometimes difficult; accuracy of pre-operative histological diagnosis of scirrhous type gastric cancer has ranged from 58% to 70% in previously published reports [13,14]. In the present case, therefore, the possibility of scirrhous type gastric cancer could not be excluded because of the similarity in gross appearance despite a biopsy specimen indicating amyloid deposits with no malignant findings. Strip biopsy was developed as an EMR technique for early gastric cancer in 1984 [15] and makes it possible to obtain a larger resected specimen compared to a biopsy specimen that enables more precise histological diagnosis. It was particularly important in this case that scirrhous-like localized gastric amyloidosis could be diagnosed by evaluating such precise histological findings from the resected specimen, thereby excluding the existence of scirrhous type gastric cancer by only using EMR without the necessity of performing an invasive surgical resection.

Primary amyloidosis refers to the disorder in patients with no preceding or co-existing disease except immunocyte dyscrasis in which the extracellular substance is composed of AL protein produced by plasma cells, as typically seen in multiple myeloma. The major forms of systemic amyloidosis also include reactive systemic amyloidosis consisting of a non-immunoglobulin protein secreted by the liver in the setting of chronic inflammatory disorders or cancers [3]. Localized gastric amyloidosis is characterized by AL type amyloid deposition in the mucosal or submucosal layer of the gastric wall [3,16]. In our patient’s case, deposition of AL type amyloid in the stomach was revealed by detailed histological examination of the EMR specimens, including Congo red staining with prior potassium permanganate incubation. The diagnosis of localized gastric amyloidosis was then confirmed after ruling out systemic amyloid involvement in other organs. Obtaining sufficient tissue specimens from representative changes in our patient’s lesion using EMR, therefore, made it possible to initially perform a detailed histological evaluation including differential staining of elastic fibers and subsequently confirm the localized deposition of AL type amyloid in the stomach. In addition, we were also able to exclude gastric malignancies such as gastric cancer and malignant lymphoma by evaluating those same specimens, so EMR could well become an important diagnostic method for determining localized gastric amyloidosis.

Currently, there are no published reports that mention in particular any specific therapy for localized gastric amyloidosis. Some reports have documented that surgical resection with lymph node dissection may be a preferable therapeutic strategy to prevent possible complications such as bleeding and obstruction [2,6]. In contrast, no further treatment is deemed to be necessary in other reports if the patient is symptom free on clinical follow-up. In addition, several reports indicate periodic controls should be scheduled to follow evolution of the disease and facilitate early recognition of multi-organ involvement [3,7]. We did not perform any treatment in this case and there were no subsequent findings of disease progression, but an evaluation involving a large number of patients should be conducted in the future to determine the proper management of localized gastric amyloidosis.

## Conclusions

In summary, this rare case of localized gastric amyloidosis was differentiated from scirrhous gastric cancer without performing invasive surgical resection as the use of EMR provided sufficient tissue specimens from the lesion to make an accurate diagnosis of localized deposition of AL type amyloid with no malignant findings possible.

## Consent

Written informed consent was obtained from the patient for publication of this case report and any accompanying images. A copy of the written consent is available for review by the Editor-in-Chief of this journal.

## Competing interests

The authors declare that they have no competing interests.

## Authors’ contributions

TK was the main contributor to the preparation of the rough draft. HS critically revised the manuscript. HT and RK was involved in histopathological analysis. SY, SN, TF, HK and IO helped to draft the manuscript. All authors read and approved the final manuscript.
